# A scoping review of nursing interventions for reducing the negative impacts of domestic violence among women

**DOI:** 10.1186/s12912-024-02453-3

**Published:** 2024-11-14

**Authors:** Iyus Yosep, Ai Mardhiyah, Helmy Hazmi, Nita Fitria, Mamat Lukman, Ahmad Yamin, Tuti Pahria

**Affiliations:** 1 Department of Mental Health, Faculty of Nursing, Universitas Padjadjaran, Sumedang, Jawa Barat Indonesia; 2 Department of Pediatric Nursing, Faculty of Nursing, Universitas Padjadjaran, Sumedang, 45363 West Java Indonesia; 3 Department of Nursing, Faculty of Medicine, University of Malaysia Sarawak, Kota Samarahan, 94300 Malaysia; 4 Department of Fundamental Nursing, Faculty of Nursing, Universitas Padjadjaran, Sumedang, 45363 West Java Indonesia; 5 Department of Community Nursing, Faculty of Nursing, Universitas Padjadjaran, Sumedang, 45363 West Java Indonesia; 6 Department of Medical-Surgical Nursing, Faculty of Nursing, Universitas Padjadjaran, Sumedang, 45363 West Java Indonesia

**Keywords:** Domestic violence, Nursing interventions, Victims, Women

## Abstract

**Background:**

Incidences of domestic violence against women are increasingly every years. Domestic violence has the negative impacts on physical problems, psychological problems, and can even cause death. Nurses have a role for providing interventions to reduce the impact of domestic violence on women.

**Aim:**

The purpose of this study is to explore methods of nursing interventions in reducing the traumatic effect of domestic violence among women.

**Method:**

This study used a scoping review method. The literature used in this study from CINAHL, PubMed, and Scopus databases. Search articles used the keywords domestic violence, impact, women, and victims. PRISMA Extension for Scoping Reviews are used for selecting articles. The inclusion criteria for the articles in this study were that the sample was female victims of sexual violence, randomized control trial or quasi-experimental research design, and last 10 years for publications period (2013–2022).

**Result:**

From three databases, we found 579 articles. After elimination-based inclusion and exclusion criteria, we found 10 articles discussing the effect of nursing interventions in reducing the impact of domestic violence on female victims. Most of the studies from USA and the range of respondents in the articles is 112–1250 respondents. The methods used in providing nursing interventions are classified into three, namely self-management programs, counseling programs, social support programs. The activities carried out in nursing interventions in the form of psychoeducation, relaxation, meditation, and also discussions about solving problems encountered. All articles show that nursing interventions are effective in reducing the impact of domestic violence on women.

**Conclusion:**

Nurses have an important role to provide comprehensive nursing care to victims of domestic violence by paying attention to various aspects, namely physical, psychological, and spiritual aspects to improve safety and comfort of patients.

**Implication for Nursing:**

This study is the basis for nurses to provide comprehensive nursing care to reduce the impact of domestic violence among women victims of domestic violence.

## Introduction

Domestic violence is an act of violence that occurs between family members in a household in the same places [1]. Domestic violence can be interpreted as an act of violence committed by a caregiver, parent or spouse. Domestic violence is all acts against someone in a household relationship that results in misery or suffering in the form of physical, sexual, psychological suffering, and also suffering in the form of neglect of the household [2]. Research conducted in 50 countries around the world shows that between 10 and 60% of women who have been married or in pairs have experienced physical violence from their partners [3]. Furthermore, this study also states that women are more likely to be attacked, injured, raped, or killed by their partners compared to other people.

The number of cases of domestic violence continues to increase every year, which can indicate that the community, especially survivors, are beginning to realize that the actions taken by perpetrators are a form of violence, so that survivors have the right to fight for their lives to be better [4]. Some data show that the number of domestic violence that occurs in several countries is quite high [5]. The highest percentage is in Timor Leste at approximately 59%, Afghanistan at 51%, and the lowest is Singapore at 6% [6]. From the 2017 Annual Records of the Indonesian Women’s National Commission, Indonesia reached 75% or 10,205 cases of domestic violence [7]. These data indicate that cases of domestic violence are still high in several countries.

Domestic violence can happen to both men and women. Based on previous survey, approximately 60% of married women from around 50 countries in the world experience physical and sexual violence [8]. The data in Bangladesh shows that women aged 15–19 years experience physical and sexual violence with a percentage of 58% [9]. The same data were obtained from the results of a study in Peru which showed that 60% of cases of sexual violence occurred in women [10]. These data indicate that women are prone to experiencing domestic violence.

Domestic violence that occurs can be in the form of physical or verbal violence. The forms of domestic violence that occur to women can be in the form of threats, harassment, and coercion, which can lead to death [11, 12]. The results of another study show that the form of domestic violence in Bangladesh is physical violence as much as 60% [13]. Verbal violence carried out in the form of humiliation, bullying, and verbal coercion. This domestic violence behavior can have a negative impact on the victim [14].

Physical and sexual violence against women, which in this case also includes domestic violence, not only has a negative impact on the physical but also on the psychological side of the victim. This is in accordance with the results of previous studies who showed that physical and sexual violence is related to psychiatric problems, such as depression, anxiety, phobias, Post Traumatic Syndrome Disorder (PTSD), suicide, and alcohol and drug abuse [6, 15]. The severity of this physical violence can also predict the level of depression in victims. The average post-traumatic stress disorder among women who experience physical abuse is quite high, ranging from 45 to 84% [16, 17].

The impact of domestic violence on women causes physical and psychological problems. Previous studies have stated that the impacts received by victims of domestic violence include physical pain and even disability, anxiety to high levels of stress which can lead to depression and mental disorders [8]. In women, domestic violence can also interfere with reproductive health, such as decreased libido and early menopause [18]. The impacts resulting from the action of domestic violence can influence each other which causes various diseases or disorders in the person who is the victim [19, 20]. If a person experiences more than one type of violence, then the impact that that person will receive is at risk of being more fatal than those who experience only one type of violence [21, 22]. If left untreated, the impact of domestic violence can lead to death.

Interventions to reduce the impact of domestic violence on women can be carried out by health services. Previous studies have shown that psychologists can be a space for victims of domestic violence to share their problems [16]. Another study showed that nurses can provide cognitive behavior interventions in reducing the incidence and negative impact of domestic violence on women [23, 24]. These efforts aim to improve the quality of life of victims of domestic violence. Nurses also act as advocates to provide a sense of security to victims of domestic violence, provide protection, and provide interventions in reducing the traumatic effects of domestic violence incidents [25]. Nurses also become facilitators in helping victims to guide the implementation of activities in a comprehensive manner and increase victims’ participation in the interventions carried out [26].

Nurses have an important role in facilitating interventions and providing counseling to address the impact of trauma on women who are victims of domestic violence. As health professionals, nurses have a comprehensive formal educational background, such as being a Registered Nurse (RN), which includes in-depth training in nursing science, mental health, and therapeutic communication [27]. Additional qualifications such as certification in trauma or domestic violence counseling further strengthen nurses’ ability to act as counselors and educators [28]. Nurses have the responsibility to conduct a thorough assessment of the victim’s physical and psychological condition [29]. Nurses must then collaborate with a multidisciplinary team to ensure that victims receive holistic care [22]. The role of nurses as counselors and educators is the role of health workers who are in direct and continuous contact with patients. Nurses can build trusting relationships and provide consistent support, which is important in trauma recovery [30]. Previous research has also shown that interventions involving nurses in counseling and education roles have been effective in improving patients’ psychological well-being [31].

Previous systematic reviews show that nurses have an important role in providing nursing care to victims of domestic violence [32]. The study was conducted by female nurses because most of the victims of domestic violence were women. The study recommends conducting future research on nursing interventions to reduce the effects of trauma on women who are victims of domestic violence. Other studies also show that nursing interventions can reduce the effects of trauma on child victims of violence [33, 34]. Nurses have a role in facilitating activities and being counselors to overcome the effects of trauma on victims [35]. So this study is the first scoping review that discusses nursing interventions to reduce the traumatic impact on women who are victims of domestic violence. Based on that, the authors interested in making a scoping review of methods in providing nursing care to reduce the traumatic effect of domestic violence that occurs in women. The purpose of this study is to explore methods of nursing interventions in reducing the traumatic effect of domestic violence among women.

## Materials and methods

### Design

The study design in this research is a scoping review. Scoping review is a design method with the aim of exploring and discussing topics that are currently developing [36]. This scoping review was chosen because it can discuss research results with a wide conceptual range based on the research objectives [37]. The stages in this study are determining research questions, determining the criteria for articles to be reviewed, selecting articles, analyzing articles, and making reports on the results of article reviews [38]. This study used PRISMA Extension for Scoping Reviews (PRISMA-ScR) to select and identify various topics that discuss nursing interventions to reduce the traumatic effect of domestic violence on women [39].

### Search methods

This study used PCC’s framework for literature searches: Population: women; Content: nursing intervention, nursing care; Context: reducing negative impact of domestic violence, using english and period of publication is last 10 years to get the new interventions to reduce negative impact of domestic violence. There are 3 databases used for literature search, namely: CINAHL, Pubmed, and Scopus. Article searches were carried out in November 1st - December 31st 2022. The keywords used are: “nursing care OR nursing intervention” AND “domestic violence OR family violence” AND “impact OR effect” AND “women OR wife”. The authors used Boolean Operator when searching the reports. In CINAHL, we use MH to specify the subject headings, which helps in capturing articles indexed under the specific topics. For PubMed, the search includes both MeSH terms and text words (tw). MeSH terms ensure the search covers articles indexed with specific medical subject headings, while text words cover the presence of terms in titles, abstracts, and other fields. In Scopus, we use TITLE-ABS-KEY to search for terms in the title, abstract, and keywords of articles.

CINAHL: ((MH “Nursing Care+”) OR (MH “Nursing Intervention+”) OR “nursing care” OR “nursing intervention” OR “nursing practice” OR “nursing management” OR “nursing treatment” OR “nursing actions”) AND ((MH “Domestic Violence+”) OR (MH “Family Violence+”) OR “domestic violence” OR “family violence” OR “intimate partner violence” OR “spousal abuse” OR “partner abuse” OR “relationship violence” OR “domestic abuse”) AND ((MH “Impact+”) OR (MH “Effect+”) OR “impact” OR “effect” OR “consequence” OR “outcome” OR “result” OR “influence”) AND ((MH “Women+”) OR (MH “Wives+”) OR “women” OR “wife” OR “female” OR “wives” OR “females” OR “spouse”).

PubMed: ((“Nursing Care“[MeSH] OR “Nursing Intervention“[MeSH] OR “nursing care“[tw] OR “nursing intervention“[tw] OR “nursing practice“[tw] OR “nursing management“[tw] OR “nursing treatment“[tw] OR “nursing actions“[tw]) AND.

(“Domestic Violence“[MeSH] OR “Family Violence“[MeSH] OR “domestic violence“[tw] OR “family violence“[tw] OR “intimate partner violence“[tw] OR “spousal abuse“[tw] OR “partner abuse“[tw] OR “relationship violence“[tw] OR “domestic abuse“[tw]) AND (“Impact“[MeSH] OR “Effect“[MeSH] OR “impact“[tw] OR “effect“[tw] OR “consequence“[tw] OR “outcome“[tw] OR “result“[tw] OR “influence“[tw]) AND (“Women“[MeSH] OR “Wives“[MeSH] OR “women“[tw] OR “wife“[tw] OR “female“[tw] OR “wives“[tw] OR “females“[tw] OR “spouse“[tw]))

Scopus: ((“Nursing Care“[MeSH] OR “Nursing Intervention“[MeSH] OR “nursing care“[tw] OR “nursing intervention“[tw] OR “nursing practice“[tw] OR “nursing management“[tw] OR “nursing treatment“[tw] OR “nursing actions“[tw]) AND.

(“Domestic Violence“[MeSH] OR “Family Violence“[MeSH] OR “domestic violence“[tw] OR “family violence“[tw] OR “intimate partner violence“[tw] OR “spousal abuse“[tw] OR “partner abuse“[tw] OR “relationship violence“[tw] OR “domestic abuse“[tw]) AND (“Impact“[MeSH] OR “Effect“[MeSH] OR “impact“[tw] OR “effect“[tw] OR “consequence“[tw] OR “outcome“[tw] OR “result“[tw] OR “influence“[tw]) AND (“Women“[MeSH] OR “Wives“[MeSH] OR “women“[tw] OR “wife“[tw] OR “female“[tw] OR “wives“[tw] OR “females“[tw] OR “spouse“[tw]))

The terms within each category are combined using OR to include synonyms and related terms. The categories are then combined using AND to ensure the search results include all relevant aspects. The research questions are: What are the methods of nursing interventions to reduce the traumatic effect of domestic violence on women?

### Inclusion and exclusion criteria

**Fig. 1:**
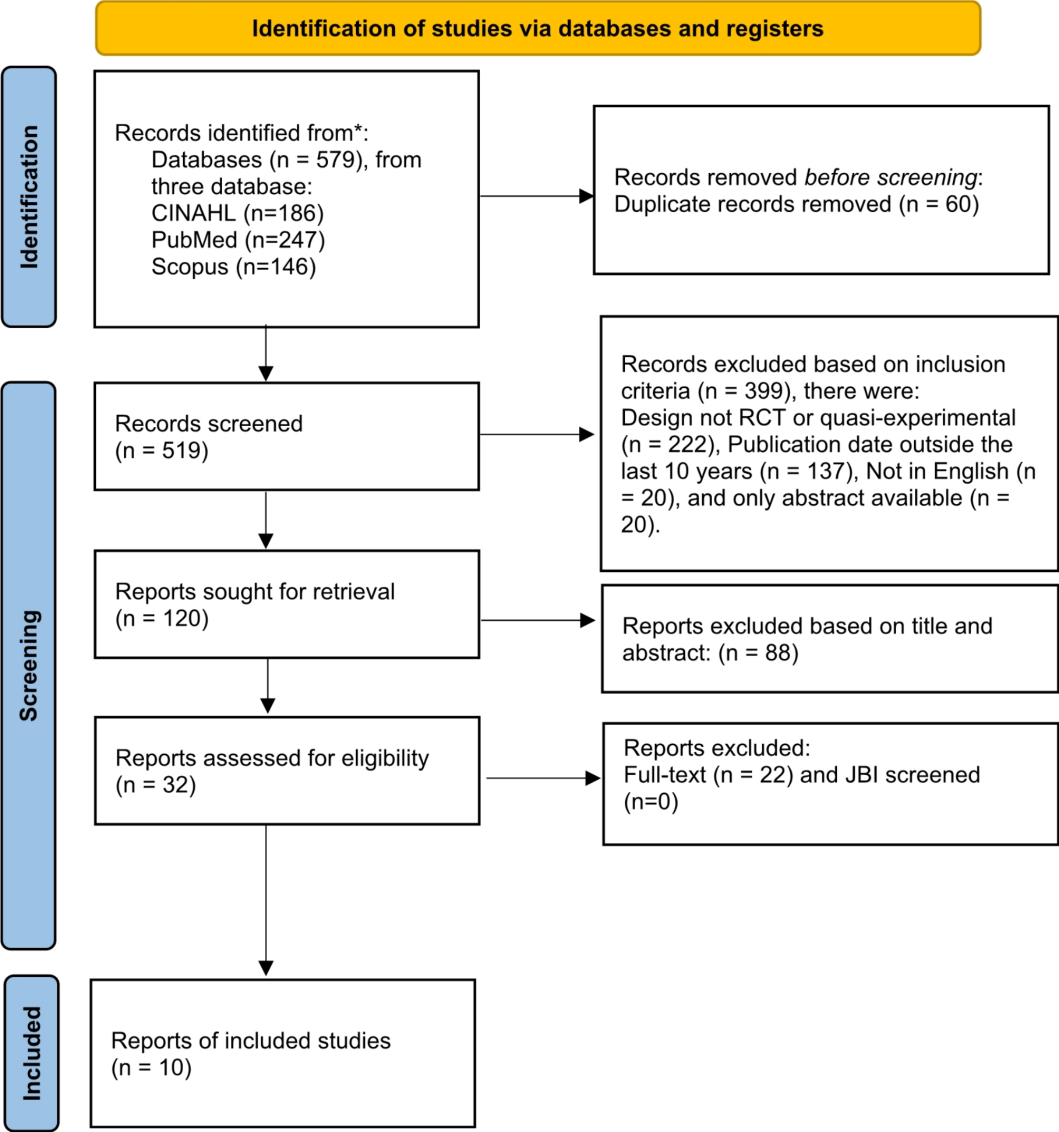
PRISMA flow diagram

This study used the PRISMA Extension for Scoping Review (PRISM-ScR) to identify types of nursing interventions that can reduce the negative impact of domestic violence on women (Fig. 1). Articles selected in the scoping review were selected based on inclusion and exclusion criteria.

The inclusion criteria for this study were carefully chosen to ensure relevance, quality, and applicability. A sample of women who experienced domestic violence was selected to address their unique needs and challenges. Limiting the language to English allowed for consistent and comprehensive analysis, while requiring full-text articles ensured access to complete data and methodologies. Interventions are carried out independently by nurses or in collaboration between nurses and other health workers and only original articles are included in this scoping review. The inclusion of randomized control trials or quasi-experimental designs guaranteed a high level of evidence, as these designs are robust in establishing causal relationships and intervention efficacy. The time setting of the last 10 years (2013–2022) was chosen to incorporate the most recent evidence and trends, ensuring that the findings are current and reflective of recent advancements in nursing care and domestic violence interventions. Meanwhile, the exclusion criteria were grey literature and only abstract.

The authors search for articles from three databases, namely CINAHL, PubMed, and Scopus. After that, the authors eliminated duplicate articles using the Mendeley application. Then all the authors analyzed the articles using inclusion criteria. The authors discussed eliminating articles by reading titles and abstracts to determine which articles are in accordance with the research objectives. Then the authors read the full-text articles that match the inclusion criteria. One article is read by three authors to discuss the contents of the articles that have been read. Data collection used manual tables was carried out after the authors double-checked the articles found.

### Data extraction

Manual tables were used by the authors to extract data. The purpose of making a table of study results is to make it easier for readers and authors of the results of the research articles being reviewed. The authors make it easier to compare and classify the data obtained. The data made in the data extraction table are the authors, year, country, research design, population and sample, procedures, interventions, and results of the study. The data extraction was conducted independently by two authors. During this process, any disagreements or differences of opinion that arose between the two authors were resolved through discussion and deliberation. If a consensus still could not be reached, a third authors was invited to extract data from the disputed reports to resolve the issue.

### Quality appraisal

The articles analyzed in this study have been assessed for quality using the critical assessment method of The Joanna Brigs Institute (JBI). JBI is an article assessment method by providing a statement on the content of the article being reviewed. JBI was chosen by the authors because it has complex statements to assess article quality based on article design. Assessment is carried out by scoring from each statement consisting of yes, no, unclear, and not applicable. The score “yes” is given a value of 1, while the other scores are given a value of zero. The standards given by the authors in this study are articles with a JBI assessment score above 75% based on criteria and topic relevance (Table 1).

**Table 1:** JBI critical appraisal tool

Author, Published Year	JBI Critical Appraisal Tool	Study Design
(Sijbrandij et al., 2016)	84.6% (11/13)	Pilot RCT
(Hegarty et al., 2013)	92.3% (12/13)	RCT
(Andersen et al., 2021)	76.9% (10/13)	RCT
(Bryant et al., 2017)	76.9% (10/13)	RCT
(Fuhr et al., 2019)	92.3% (12/13)	RCT
(Mutisya et al., 2018)	88.9% (8/9)	Quasi-experimental
(Sabri et al., 2019)	84.6% (11/13)	RCT
(Patel et al., 2017)	92.3% (12/13)	RCT
(Bass et al., 2016)	76.9% (10/13)	RCT
(Hansen et al., 2014)	76.9% (10/13)	RCT

The JBI assessment questions for the Quasi experimental design consist of 9 questions, namely Is it clear in the study what is the “cause” and what is the “effect” (i.e. is there no confusion about which variable comes first)?; Was there a control group?; Were participants included in any similar comparisons?; Were the participants included in any comparisons receiving similar treatment/care, other than the exposure or intervention of interest?; Were there multiple measurements of the outcome, both pre and post the intervention/exposure?; Were the outcomes of participants included in any comparisons measured in the same way?; Are outcomes measured in a reliable way?; Was follow-up complete and if not, were differences between groups in terms of their follow-up thoroughly described and analyzed?; and Was appropriate statistical analysis used?. In this study, there was one question that was not met, namely that the group did not receive intervention similar to the experimental group.

The JBI assessment questions for the Randomized control trial design, consist of 13 questions, namely Was true randomization used for assignment of participants to treatment groups?; Was allocation to treatment groups concealed?; Were treatment groups similar at the baseline?; Were participants blind to treatment assignment?; Were those delivering treatment blind to treatment assignment?; Are outcomes assessors blind to treatment assignment?; Are treatment groups treated identically other than the intervention of interest?; Was follow up complete and if not, were differences between groups in terms of their follow up thoroughly described and analyzed?; Were participants analyzed in the groups to which they were randomized?; Are outcomes measured in the same way as treatment groups?; Are outcomes measured in a reliable way?; Was appropriate statistical analysis used?; and Was the trial design appropriate, and any deviations from the standard RCT design (individual randomization, parallel groups) accounted for in the conduct and analysis of the trial? In this study, most of the studies used a randomized control trial design. Each study had yes answers in the range of 10–12 questions. The questions that do not have a yes answer are that the blinded process was not explained in writing by the authors. Such as the question “Were participants blind to treatment assignment?; Were those delivering treatment blind to treatment assignment?; Were outcomes assessors blind to treatment assignment?” was not explained in writing by the authors. While other questions were explained in writing by the authors.

### Data analysis

Data were analyzed by descriptive approach. Articles that have been eliminated based on inclusion and exclusion criteria and have complied with the standards of article quality assessment are then read in full and analyzed by all authors. The authors then create a manual table that contains data from the reviewed articles. After that, the authors make a description of the nursing interventions obtained from the results of the study. The description of the study results was made by classifying similar nursing interventions based on the results of discussions between all authors. The data analysis was performed systematically to ensure accuracy and comprehensiveness. Initially, the two authors independently reviewed and analyzed the extracted data. Any discrepancies or differences in their analyses were addressed through thorough discussion and consensus-building. In cases where a consensus could not be reached, a third authors was consulted to provide an additional perspective and help resolve the differences. This methodical approach to data analysis ensured that the findings were robust and reliable, contributing to the overall quality and credibility of the review.

## Results

The authors found 579 articles from article search results used three databases. Then the authors checked for duplication of articles using the Mendeley application, the authors get 60 duplicate articles. Of the 519 articles, then the authors eliminated articles by reading titles and abstracts and screening based on inclusion criteria, The authors found that there were 487 articles that did not meet the criteria. After the authors read and analyze the 32 articles, the authors get 10 articles that match the criteria and are relevant to the purpose of this study. To ensure that the article is appropriate, the authors double-checked. Then the authors checked the quality of the article using the JBI assessment instrument. The authors determine that the standard of the article used in this study is to have a JBI score above 75% (Table 1).

There were 10 articles who discussed about nursing intervention to reduce the negative impact on women as victim of domestic violence. Nursing interventions are carried out mostly in developed countries. Articles originating from developed countries, namely 3 articles from the United States of America, 1 article from the Netherlands, 1 article from Australia, 1 article from Kenya, 1 article from the United Kingdom, and 1 article from Denmark. while articles originating from developing countries, namely 1 article from India and 1 article from Iraq. Most of the interventions are carried out in the USA (Table 2). The largest sample which is a woman also comes from the USA with a total sample of 1250. Each nursing intervention requires consideration of the patient’s condition to reduce the impact of domestic violence.

**Table 2:** Characteristic of study

Country	Authors and Year
Netherlands	(Sijbrandij et al., 2016)
Australia	(Hegarty et al., 2013)
USA	(Andersen et al., 2021; Bryant et al., 2017; Sabri et al., 2019)
India	(Fuhr et al., 2019)
Kenya	(Mutisya et al., 2018)
United Kingdom	(Patel et al., 2017)
Iraq	(Bass et al., 2016)
Denmark	(Hansen et al., 2014)
**Design**	
RCT	(Andersen et al., 2021; Bass et al., 2016; Bryant et al., 2017; Fuhr et al., 2019; Hansen et al., 2014; Hegarty et al., 2013; Patel et al., 2017; Sabri et al., 2019)
Pilot RCT	(Sijbrandij et al., 2016)
Quasi experiment	(Mutisya et al., 2018)

The results of the analysis of the article are presented in manual form from all authors presented as follow (Table 3):

**Table 3:** The explanation of each nursing intervention method

Self Management				
Authors and Year	Purpose	Sample Size	Method	Result
(Sijbrandij et al., 2016)	Effect PM + on women who have domestic violence	494	Problem Management Plus (PM+): PM + integrates problem-solving and behavioral techniques to address stress, problem-solving, and social support. It is delivered in five weekly sessions of 90 min, focusing on stress management, problem-solving, behavioral activation, and strengthening social support.	Significant on reduce psychological distress, functional disability, and PTSD symptoms
(Bryant et al., 2017)	effectiveness of behavioral interventions in psychological distress	518	Behavioral Treatment Called PM+ (Home-Based): This version of PM + includes five sessions delivered at home, addressing stress management, problem-solving, behavioral activation, and strengthening social supports. Sessions are rescheduled if missed, with a three-attempt limit for follow-ups.	Effective to reduce psychological distress
(Andersen et al., 2021)	effective mindfulness to psychological and somatic symptoms;	112	Mindfulness-Based Stress Reduction (MBSR): MBSR is an 8-week course, with weekly 2-hour sessions, designed to teach mindfulness techniques such as breathing awareness, body scanning, and yoga. Participants engage in weekly homework and a half-day silent retreat to deepen their practice.	effective to improbe psychological and somatic symptoms
(Patel et al., 2017)	assessed the effectiveness in depressive symptoms	495	Healthy Activity Program (HAP): HAP consists of 6–8 individual 30–40 min sessions, typically delivered face-to-face or via phone. It focuses on core intervention strategies for improving mental health, with a middle phase for teaching, followed by review and termination.	Effective to reduce depressive symptoms
**Counseling Program**				
(Hegarty et al., 2013)	identify whether brief counselling to improve quality of live and behavior	272	Whether Brief Counselling: This training involves a 6-hour distance learning package and two 1-hour interactive practice visits, emphasizing patient-centered care. It includes active listening, motivational interviewing, and problem-solving techniques to support women’s decisions.	Significant to improve quality of life and reduce depressive symptoms.
(Sabri et al., 2019)	Effect of web-based intervention to reduce post stress and depressive	1250	Web-Based weWomen/ourCircle Intervention: A culturally specific, web-based intervention that helps women assess relationship danger and create a personalized safety plan based on their unique circumstances. They can access the intervention via a secure website throughout a 1-year follow-up period.	Significant to reduce post stress and depressive symptoms
**Social Supports Program**				
(Fuhr et al., 2019)	Assess effectiveness the intervention in stress and depression	140	Thinking Healthy Programme (THPP): THPP is a peer-delivered behavioral activation intervention for maternal mental health. It consists of 6–14 sessions, delivered over four phases (prenatal to late infancy) across 7–12 months, with a focus on promoting mental health during and after pregnancy.	had a moderate effect on reduce symptom of stress and depression
(Mutisya et al., 2018)	assessed the effect of a psychosocial intervention on depressive symptoms	288	Psychosocial Intervention: This intervention provides participants with resource cards for seeking help and includes three psychosocial support sessions spread over 4–5 months, coinciding with antenatal care appointments. Each session lasts 30–35 min, offering support and counselling.	Significant to improve psychological support and reduce depressive
(Bass et al., 2016)	To evaluated the interventions on post-traumatic stress, traumatic grief, and anxiety symptoms	159	Trauma-Informed Support, Skills, and Psychoeducation Intervention: This intervention involves 6–12 sessions designed for trauma survivors. It includes various techniques aimed at building a therapeutic relationship and supporting recovery from trauma, led by trained community mental health workers.	Significant to reduce the traumatic experience, post traumatic stress, and anxiety symptoms
(Hansen et al., 2014)	effects on psychological symptoms and perceived social support	212	Specific Three-Phase Intervention Program: This group therapy program consists of 14 weekly 3-hour sessions for women affected by trauma. It covers processing traumatic experiences, addressing psychological symptoms, discussing the impact on children, and planning for the future, led by a psychologist and social worker.	Significant to improve perceived social support and reduce psychological symptoms such as PTSD and depressive

We have read and analyzed 10 articles, and we are classified the intervention with three programs, there were self-management programs, counseling programs, social support programs. The authors classify intervention methods based on similar interventions performed by nurses. Then the authors describe the intervention based on the similarity of the methods.

### Self-management

Self-management is important for dealing with traumatic experiences. One of the interventions that can be done is Problem Management Plus (PM+) which is carried out for 2 weeks [40]. The interventions carried out in the form of reducing psychological distress, functional disability, PTSD symptoms, problems felt by people seeking help, use of health care, and health costs. Evaluation of the implementation of the intervention was carried out by means of an interview process. The results of the intervention showed a decrease in the impact of domestic violence.

A similar intervention, namely a behavioral treatment called Problem Management Plus (PM+) can be carried out in 6 sessions for 6 months [41]. Therapy is done with psychoeducation, problem solving strategies, stress management, and strengthening social support. The results of the intervention showed that there was a reduction in psychological distress. Another program that can be carried out is the Mindfulness-Based Stress Reduction intervention which is carried out for 8 weeks [42]. Activities carried out in the form of psychoeducation, mindfulness therapy, and strengthening coping to overcome problems. The intervention proved effective in modifying psychological and somatic symptoms.

Another self-management intervention that can be carried out is the Healthy Activity Program which performs 6 aspects, namely psychoeducation, behavior assessment, activity monitoring, activity planning and scheduling, social network activation, and problem solving [43]. The program is conducted over a period of 6 weeks with additional strategies to improve interpersonal communication skills and reduce rumination, advice on sleep problems and tobacco cessation, and relaxation training. This intervention has been shown to be effective in reducing depression and other mental health disorders.

### Counseling

Counseling is an effort to facilitate women as victims of bullying to consult about problems and problem solving strategies. The intervention that can be done is whether brief counseling is an intervention carried out in 6 sessions for 6 months [44]. The activities carried out are education about the safety of women and children, and providing supportive counseling for women who experience abuse. Furthermore, counseling is carried out regarding finding solutions to the problems faced by women victims of domestic violence. Counseling has been shown to be effective in reducing depressive symptoms.

Similar interventions that can be done, namely the web-based weWomen/ourCircle intervention, carried out for 1 year with 12 sessions [16]. The training focuses on aspects of commitment to relationships, getting community support, having resources, security, health and well-being or the welfare of their children. Participants access the web-based weWomen/ourCircle. These interventions may not only reduce the risk of victimization of violence, but also empower abused women and improve their mental health.

### Social supports

Social support is also an important intervention in reducing the impact of domestic violence on women. The intervention given in the form of The Thinking Healthy Program was carried out for 6–14 individual sessions for 4 months [45]. The intervention focuses on improving the individual’s ability to deal with problems. This intervention consists of 3 stages, namely psychoeducation, problem solving, and mindfulness. The results of the intervention show that The Thinking Healthy Program can reduce the incidence of violence against women and reduce the impact of domestic violence on women.

The psychosocial intervention consisted of three psychosocial support sessions that were conducted for 30–35 min [46]. Activities carried out in the form of psychoeducation, social support exercises, and peer support. This activity aims to improve the psychosocial abilities of victims of domestic violence. The results of the intervention showed a decrease in the negative impact of domestic violence on women.

The Trauma-Informed Support, Skills, and Psychoeducation Intervention was conducted for 6–12 sessions [47]. The number of sessions for each intervention is adjusted to the client’s needs. Programs in the form of education related to mental health, psychosocial support, and trauma psychoeducation. This intervention is effective to improve social relations and reduce mental health problems in women victims of domestic violence.

The specific three-phase intervention program is carried out in 3 phases, namely psychoeducation, relaxation techniques, and social support [48]. This intervention was carried out for 4 months to focus on the mental health of the patient. Intervention was also carried out by involving the patient’s family members. The results of this study indicate that there is a decrease in mental health problems in women who are victims of domestic violence.

## Discussion

We found 10 articles discussing nursing interventions in reducing the impact of domestic violence on women. From the 10 articles, we classify them into 3 types of intervention based on the similarity of the program, there are self-management programs, counseling programs, and social support programs. Nurses have an important role to provide education, training, and counselors for every program that is run. Nurses can also become facilitators to assist participants in carrying out their activities.

The results of this study indicate that nursing interventions can be carried out for 3–6 months. In line with previous studies which show that the time to reduce the impact of domestic violence is 3 months. This is because the impact of domestic violence causes a high sense of trauma which causes women’s mental health problems to decrease [8]. The results of previous studies stated that domestic violence can cause lasting traumatic effects on women as victims [20, 49]. Another factor that causes the length of nursing intervention is the severity of the traumatic events experienced by women as victims of domestic violence [18]. Victims need a long time to reconcile and accept the traumatic events they experienced [50]. So that the intervention process takes quite a long time to reduce the impact of domestic violence on women.

Nursing interventions in the results of this study were carried out in developed and developing countries. Incidents of domestic violence do not only occur in developing countries that have less economies. Although many incidents of domestic violence are caused by low family economic conditions [7]. Possible causes of domestic violence in developed countries is an affair. This is in line with previous studies which show that extramarital affairs in developed countries lead to domestic violence in women [51]. Meanwhile, other studies in developing countries show that the cause of domestic violence in women is economic factors [52].

In some societies, norms that permit or even promote violence as a way to resolve conflict can increase the risk of domestic violence [53]. Educational factors also influence domestic violence, as individuals with lower levels of education tend to have less understanding of their rights and ways to resolve conflict without violence [25]. In addition, individuals who grow up in environments that witness or experience violence are likely to repeat the pattern in their own relationships [54]. In developing countries it can also lead to difficulties in accessing support and protection services in the community. Individuals who do not have access to these resources may feel trapped in violent situations with no way out [55].

Nurses have a role to provide comprehensive nursing care covering all aspects, namely physical, psychological, social, spiritual, and cultural aspects. Nurses act as counselors, facilitators, advocates, and educators. In line with previous studies which show that nurses have an important role as advocates to protect women who experience domestic violence [56, 57]. So that victims feel safe and comfortable in carrying out nursing interventions. Nurses also have a role as counselors to assist victims in overcoming the problems and effects of trauma due to domestic violence. Previous study have shown that interventions carried out by nurses for victims of domestic violence can increase patient comfort because they are given comprehensive nursing care covering all aspects that affect victims [11, 58].

Nursing interventions that can be carried out are self-management programs. Self-management is an intervention to manage the patient’s self to live life in accordance with predetermined goals. The impact of domestic violence on women causes mental health problems, making it difficult to control oneself in doing things [59]. Good self-management in victims can control their emotions so that they are able to use adaptive coping in responding to stressors. Adaptive coping helps victims recover from the traumatic experiences they experience. Other studies show that self-management can reduce the traumatic impact of violence [13, 15]. In addition, self-management interventions can also increase the productivity of victims of violence.

Counseling programs are one of the interventions that can be carried out to reduce the impact of domestic violence on women. Women who experience domestic violence are usually difficult to express the traumatic experiences they experience [60]. So that victims need space to make peace and accept their traumatic events. Previous studies have shown that nursing interventions with counseling can reduce traumatic feelings and improve mental health experienced by women who experience domestic violence [61–63]. Counseling is carried out by nurses with psychologists to express the feelings of victims of domestic violence.

Counseling service activities for women victims of domestic violence are assistance provided by counselors for professional counseling to solve problems of psychological disorders and emotional traumas experienced as a result of domestic violence. Counseling services aim for women to be able to overcome their own difficulties through the counseling process carried out so that women can carry out their daily activities optimally [62, 63]. This is in line with previous studies which show that counseling conducted by nurses for women victims of domestic violence can reduce the effects of trauma on victims. The things that must be considered in providing counseling are carried out by female nurses. Female nurses who provide counseling can create a safe space and comfort for victims of domestic violence. This is in line with previous studies which show that female health workers can increase the success of counseling programs that occur in women who have had traumatic experiences [64, 65].

Another intervention that can be done to reduce the impact of domestic violence on women is social support programs. This intervention focuses on social support for victims of domestic violence. The impact of domestic violence causes victims to experience social isolation and decreased self-confidence [14]. So it is difficult to communicate with other people. Social support will increase the self-confidence of victims of domestic violence [66]. Social support will make women who experience domestic violence believe that they are not facing their problems alone. This is supported by previous studies which showed an increase in self-esteem in victims of domestic violence who were given social support interventions [7, 11, 67]. Social support will improve self-confidence, mental health, and the ability to communicate with others [68]. The implementation of social support activities involves women, teachers and health workers to form peer-groups that support each other in the intervention process to heal the effects of trauma on victims of domestic violence.

Social support is defined as an action carried out by people around the victim who can provide resources, such as emotional, information, in the form of support needed by the victim. Several previous studies have shown that there is a relationship between the role of social support and psychological disorders (depression and post-traumatic stress disorder). Other studies also show results that the low support that victims get from family or friends around them exacerbates depression and anxiety experienced by victims [69, 70]. Meanwhile, the existence of social support can relieve the physical and psychological pressure experienced and enable victims to cope with the pressure experienced better than individuals who do not get social support from those around them. The existence of social support that is immediately given makes women as victims of domestic violence able to choose and adopt positive ways of coping or solving problems and reduce the effects of trauma due to domestic violence [55, 71, 72].

Nursing interventions are important to be given to women victims of domestic violence. The provision of comprehensive nursing care increases the safety and comfort of the victim so as to provide protection to the victim. In addition, nurses also pay attention to various aspects that affect trauma to victims of domestic violence so as to significantly reduce the effects of trauma. This is in line with previous studies which show that nursing interventions given intensively can significantly reduce the impact of trauma on children who experience violence [73–75].

Nursing intervention is an effective way to reduce the impact of domestic violence on women. In its implementation, it is necessary to pay attention to aspects from the victim’s side. The facilitator must side with the victim and see the victim’s point of view. So that interventions are carried out according to the conditions experienced by victims of domestic violence.

### Implication for nursing

The implication of this study is that it can serve as a basis for nurses to intervene on female victims who experience domestic violence. And can be an illustration for health services in promoting nursing interventions in reducing the impact of domestic violence.

### Limitations

The limitation in this study is the publication time of articles in the scoping review is limited to the last 10 years. This causes the authors to be unable to comprehensively discuss data regarding nursing interventions carried out more than 10 years ago in reducing the impact of domestic violence on women. The research design in the article is also limited, namely RCT and quasi-experimental, so there is no qualitative supporting data indicating a significant increase in the interventions provided. Interventions are also limited to being carried out by nurses, so there are no other options in carrying out interventions that can be carried out by other health workers or independently by the family.

## Conclusion

This study shows that there are 10 articles that describe nursing interventions in reducing the impact of domestic violence on female victims. Nurses have an important role as educators and counselors for victims of domestic violence. Nursing interventions have effectiveness in reducing the impact of domestic violence through three methods, namely self-management programs, counseling programs, and social support programs. The activities carried out in the form of psychoeducation, meditation and relaxation, and strategies to improve coping and problem solving. Suggestion for future study is the need for research on the effectiveness of nursing interventions in women who experience domestic violence in developing countries.

## Data Availability

All data generated or analysed during this study are included in this published article.
